# Clinical and public health implications of acute and early HIV detection and treatment: a scoping review

**DOI:** 10.7448/IAS.20.1.21579

**Published:** 2017-06-28

**Authors:** Sarah E. Rutstein, Jintanat Ananworanich, Sarah Fidler, Cheryl Johnson, Eduard J. Sanders, Omar Sued, Asier Saez-Cirion, Christopher D. Pilcher, Christophe Fraser, Myron S. Cohen, Marco Vitoria, Meg Doherty, Joseph D. Tucker

**Affiliations:** ^a^ Department of Health Policy and Management, University of North Carolina at Chapel Hill, Chapel Hill, NC, USA; ^b^ Division of Infectious Diseases, University of North Carolina at Chapel Hill, Chapel Hill, NC, USA; ^c^ U.S. Military HIV Research Program, Walter Reed Army Institute of Research, Silver Spring, MD, USA; ^d^ Henry M. Jackson Foundation for the Advancement of Military Medicine, Bethesda, MD, USA; ^e^ Department of Medicine, Imperial College London, London, UK; ^f^ HIV Department, World Health Organization, Geneva, Switzerland; ^g^ Department of Clinical Research, London School of Hygiene and Tropical Medicine, London, UK; ^h^ Department of Global Health, University of Amsterdam, Amsterdam, The Netherlands; ^i^ Kenya Medical Research Institute-Wellcome Trust Research Programme, Kilifi, Kenya; ^j^ Nuffield Department of Medicine, University of Oxford, Oxford, UK; ^k^ Fundación Huésped, Buenos Aires, Argentina; ^l^ Institut Pasteur, HIV Inflammation and Persistance Unit, Paris, France; ^m^ HIV/AIDS Division, University of California San Francisco, San Francisco, CA, USA; ^n^ Big Data Institute, Nuffield Department of Medicine, University of Oxford, Oxford, UK; ^o^ Department of Epidemiology, University of North Carolina at Chapel Hill, Chapel Hill, NC, USA; ^p^ UNC Project-China, Institute for Global Health and Infectious Diseases, University of North Carolina at Chapel Hill, Chapel Hill, NC, USA

**Keywords:** acute HIV infection, antiretroviral therapy, diagnostics, early HIV, guidelines, low- and middle-income countries, primary HIV

## Abstract

**Introduction**: The unchanged global HIV incidence may be related to ignoring acute HIV infection (AHI). This scoping review examines diagnostic, clinical, and public health implications of identifying and treating persons with AHI.

**Methods**: We searched PubMed, in addition to hand-review of key journals identifying research pertaining to AHI detection and treatment. We focused on the relative contribution of AHI to transmission and the diagnostic, clinical, and public health implications. We prioritized research from low- and middle-income countries (LMICs) published in the last fifteen years.

**Results and Discussion**: Extensive AHI research and limited routine AHI detection and treatment have begun in LMIC. Diagnostic challenges include ease-of-use, suitability for application and distribution in LMIC, and throughput for high-volume testing. Risk score algorithms have been used in LMIC to screen for AHI among individuals with behavioural and clinical characteristics more often associated with AHI. However, algorithms have not been implemented outside research settings. From a clinical perspective, there are substantial immunological and virological benefits to identifying and treating persons with AHI – evading the irreversible damage to host immune systems and seeding of viral reservoirs that occurs during untreated acute infection. The therapeutic benefits require rapid initiation of antiretrovirals, a logistical challenge in the absence of point-of-care testing. From a public health perspective, AHI diagnosis and treatment is critical to: decrease transmission via viral load reduction and behavioural interventions; improve pre-exposure prophylaxis outcomes by avoiding treatment initiation for HIV-seronegative persons with AHI; and, enhance partner services via notification for persons recently exposed or likely transmitting.

**Conclusions**: There are undeniable clinical and public health benefits to AHI detection and treatment, but also substantial diagnostic and logistical barriers to implementation and scale-up. Effective early ART initiation may be critical for HIV eradication efforts, but widespread use in LMIC requires simple and accurate diagnostic tools. Implementation research is critical to facilitate sustainable integration of AHI detection and treatment into existing health systems and will be essential for prospective evaluation of testing algorithms, point-of-care diagnostics, and efficacious and effective first-line regimens.

## Introduction

Despite comprehensive HIV interventions and scaled-up antiretroviral therapy (ART) services in many regions, there are still two million new adult HIV cases each year [1]. Failure of existing prevention initiatives, in which many persons with HIV are not on ART nor virally suppressed, may explain some of the ongoing transmission events. However, the largely unchanged HIV incidence must in part be the result of current programmes failing to detect and treat acute HIV infection [2]. According to the original Fiebig classification [3], acute infection is defined as the period between viral acquisition and emergence of HIV-specific antibodies, generally accompanied by a burst of viremia [4]. By comparison, early infection, inclusive of the acute phase, represents an approximately 6-month period of increased transmission efficiency [5–9].People with acute or early infection (hereafter referred to as AHI) likely play a disproportionate role in HIV transmission [6,7]; heightened efforts to identify and manage persons with AHI are critical to decrease incidence.

Despite the importance of AHI, optimal clinical and public health strategies for AHI are unclear. The US Department of Health and Human Services HIV guidelines recommend universal screening and treatment of AHI and the European AIDS Clinical Society also recommends universal treatment for primary HIV infection [10–12]. Although the International AIDS Society and the World Health Organization’s (WHO) most recent guidelines support scale-up of testing and immediate treatment for all people diagnosed with HIV, there is no specific recommendation regarding diagnostic strategies or ART for persons with AHI [13–16]. From a public health standpoint, undiagnosed AHI could interfere with the extent to which comprehensive strategies such as *Treat All* could effectively achieve desired population health benefits. Undiagnosed AHI may also compromise the effectiveness of pre-exposure prophylaxis (PrEP), increasing the risk of resistant virus if PrEP is initiated [15,17]. Early treatment initiation is required to avert irreversible damage to the immune system [18], and to shrink the latent reservoir that renders HIV incurable [19,20]

A public health approach to AHI is necessary, especially in resource-constrained low- and middle-income countries (LMICs). The case for prioritizing resources to detect and treat persons with AHI demands a thorough evaluation of the state of knowledge and projected impact of AHI on new and established HIV epidemics. Previous AHI reviews have focused on basic science [19,21], concentrated epidemics in high-income countries [22–25], and programmatic considerations [26,27]. The purpose of this scoping review is to examine the diagnostic, clinical, and public health implications of identifying and treating persons with AHI, especially in LMIC settings.

## Methods

We conducted a scoping review [28] synthesizing the most recent literature on clinical and public health implications of widespread AHI detection and treatment. This review includes clinical trials, observational studies, systematic and non-systematic reviews, mathematical modelling, and best practice guidelines. Scoping reviews are distinct from systematic reviews in the absence of a priori article criteria; although we emphasized research published in the last 15 years, there were no explicit inclusion nor exclusion criteria for articles. Instead, this included papers that map the current state of research, identifying gaps in knowledge [29]. We examined a broad range of literature and topics relevant to the clinical and public health effects of under-diagnosed and under-treated AHI. Based on the scoping review, we summarize key policy implications and areas for research priorities.

We identified studies using keyword searches in PubMed (last searched 8 October 2016), expert opinion, and current regional and international HIV testing and treatment guidelines. For database searches, we used phrases and synonymous variations of the following terms: acute HIV, primary HIV, early HIV, treatment, and diagnosis. We also identified studies based on searches of reference lists, hand-searching key journals identified from initial database inquiries, and unpublished conference abstracts. We prioritized inclusion of LMIC research. Our search included studies that provided empirical data on AHI published in the last fifteen years. We included studies that had the following elements: (1) focus on AHI detection, treatment, or public health features (i.e., algorithms to detect AHI, implications of AHI on PrEP); (2) provide empirical data on AHI testing or treatment outcomes; (3) evaluate natural history of AHI as it relates to morbidity and transmission; or (4) model the impact or role of AHI in emerging or established epidemics.

## Results and discussion

### Impact of AHI on secondary transmission

Data from clinical studies, phylogenetic research, and modelling support the importance of AHI in HIV transmission in LMIC as well as wealthy nations among both MSM and heterosexually-dominated epidemics. Highly infectious founder viruses [30] coupled with 10-fold higher viral loads [3,5–8,31,32] suggest there is a greater likelihood of transmission during AHI.

Empirical data estimating HIV transmission rates are sparse. The Rakai study is one of the largest prospective cohort studies to investigate transmission rates, enrolling greater than 15,000 adults from the Rakai district in Uganda and identifying over 400 serodiscordant couples for assessment of transmission events and the associated risk of transmission by stage of infection of the index participant [33]. Rakai data have been evaluated three separate times with widely different estimates for the extent to which AHI accounts for onward transmission among heterosexual serodiscordant couples (Table 1) [34–36]. These differences can be ascribed to adjustments in estimated duration of infectious stages; assumptions regarding determination of transmission hazards (i.e. reliance on self-reported coital frequency, as a function of time, or using predicted viral load trajectories); accounting for diversity in both infectiousness and susceptibility; and modification to the initial analyses’ couple exclusion criteria. Importantly, the proportion of transmissions attributable to AHI is likely to change with increased ART coverage – with more HIV-infected persons virally suppressed on ART, and thus less likely to transmit, persons with AHI may become an increasingly important population to target to reduce incidence.

**Table 1.** Variability in estimated HIV-1 transmission risk during AHI vs. chronic infection as evaluated using Rakai-based HIV-discordant couple cohort

Author, year	AHI definition (months)	Analytic methods	Assumptions	Outcomes: Increased infectiousness, AHI vs. chronic infection
Wawer et al.[34]	0–5	Estimated per coital transmission rates by index partner infection stage	Relied on self-reported coital frequency Stages of infectivity defined by 10-month interval observation periods	12-fold Per coital act: 0.0082 vs. 0.0007
Hollingsworth et al.[35]	0–3	Probabilistic model estimated transmission hazards by infection stage	Adjusted duration of infectious stages based on predicted infectiousness Transmission hazard as a function of time since partnership formed (i.e. not transmissions/reported coital acts)	26-fold Transmission hazard/100 person-years: 276 vs. 10.6
Bellan et al.[36]	0–1.7	Simulated cohort model, fitting Rakai-population transmission events based on transmission hazard and AHI duration using Bayesian methods	Used observed VL trajectories and predicted VL-infectivity relationship to estimate infectiousness Adjusted for heterogeneity of infectiousness and susceptibility Modified original analyses’ exclusion criteria to include previously censored couples^a^	5-fold Median transmission hazard/year: 0.62 vs. 0.12

^a^Original cohort censorship of serodiscordant couples based on single study visits prior to (a) being lost to follow-up, (b) couple dissolution, or (c) study termination.

AHI – acute HIV infection; VL – viral load.

#### Modelling AHI’s role in epidemics

Phylogenetic research has demonstrated the importance of AHI in HIV epidemics. Phylogenetic studies show that persons with recent infection drive transmission clusters; however, estimates are highly variable, identifying persons with AHI as source infection for between 10% and 50% of all transmissions [22,37–47]. Discrepancies are due to differences in epidemic stage, definitions of AHI, and variation in risk behaviours (i.e., anal vs. vaginal intercourse) by region and population [48]. For example, partner-change patterns are likely variable across regions and sub-populations, thus estimates derived from European men who have sex with men (MSM) may not be generalizable to heterosexual transmission in sub-Saharan Africa.

Mathematical models largely agree that persons with AHI are responsible for a disproportionate share of all transmissions, although there is significant variability in estimates [34,49–54]. Models based on data from Malawi estimated that approximately 40% of transmissions were attributable to persons with AHI [9], similar to results from the US among MSM [54]. However, models populated using data from elsewhere in sub-Saharan Africa predict AHI-attributable transmissions <20% [55], or as low as 3% [56]. Similar to the discrepancies across phylogenetic studies, differences in mathematical model estimates can be traced to variation in model design and input assumptions. Ongoing population-based studies, including PopART [57] and SEARCH [58], will help to better parameterize models.

### Diagnosing AHI

#### AHI diagnostics

Diagnosing AHI is complicated by its brief duration and absence of detectable antibodies. Third-generation HIV rapid diagnostic tests (RDTs), the backbone of testing in most LMICs [14], only detect HIV-specific antibodies, missing the earliest pre-antibody phase (Table 2). Fourth-generation antigen-antibody combination enzyme immunoassays (EIA) shorten the seroconversion detection window, detecting both HIV-1/2 antibodies and p24 antigens [59–64], and detects the majority of persons with AHI when applied after a negative 3rd generation test [65]. Fourth-generation tests are now standard in Europe, the USA, and parts of Latin America [66,67], but scale up may be more difficult in other LMIC where the majority of testing takes place in lower-tier health facilities. Although experience with 4th generation EIAs in LMICs has been largely restricted to research study settings, results are encouraging [68]. Nonetheless, compared to RNA testing, 4th generation assays miss patients in the earliest stages of AHI (prior to detectable p24 antigen [69]) – precisely the group that some studies suggest may benefit most from immediate treatment [19]. Fourth-generation tests may also be falsely negative in the setting of early treatment of AHI: one Thai study found that 17% of patients retested with 4^th^ generation assays after treatment during AHI either failed to convert or reverted to nonreactivity, compared with 4% for 3^rd^ generation tests [70]. Pilot testing of 4^th^ generation EIAs and NAT such as the ongoing RV254/SEARCH010 cohort study [71] will be critical to better understand more widespread use of this technology.

**Table 2.** HIV diagnostics in the context of AHI

Test	Advantages	Disadvantages	*Cost* (per test, USD)^b^
3^rd^ generation antibody (POC)	ASSURED criteria: Affordable, sensitive, specific, user-friendly, rapid & robust, equipment free, delivered	Misses earliest phase of infection	1.5
4^th^ generation antibody/antigen (EIA)	Detects infection earlier with p24 antigen sensitivity	Requires skilled personnel for specimen collection/laboratory processing Does not discriminate between antigen and antibody assay targets (i.e., acute and established HIV) without modified signal-to-cutoff ratios^Ŧ^ [72] Delayed result delivery increases risk of loss-to-follow-up Misses pre-p24 “eclipse” period May be prohibitively expensive for LMIC	5
4^th^ generation (POC)	ASSURED criteria: Affordable, sensitive, specific, user-friendly, rapid & robust, equipment free, delivered Narrows window period for AHI diagnosis vs. 3^rd^ generation	Poor field-performance to-date Misses pre-p24 “eclipse” period	Not available
Nucleic acid amplification testing (NAT) technologies^a^	Highly sensitive, capable of diagnosing shortly after acquisition Pooling may decrease per-test costs in appropriate ratios	Requires skilled personnel for specimen collection/laboratory processing May be prohibitively expensive for LMIC; trade-off of increasing complexity with decreasing cost for pooling strategies Delayed result delivery increases risk of loss-to-follow-up	8–10
NAT technology (POC)	ASSURED criteria: Affordable, sensitive, specific, user-friendly, rapid & robust, equipment free, delivered Highly sensitive, capable of diagnosing shortly after acquisition	Early in development – in need of additional field testing Concern for throughput feasibility with current technologies Unknown cost	17–25

^a^with and without pooling;

^Ŧ^Ramos et al. propose a modified testing algorithm through which reduced signal-to-cutoff ratios would trigger confirmatory Multispot and NAT testing to increase sensitivity of 4^th^ generation testing in detecting AHI. This ratio is determined using the signal strength of a sample compared to the signal strength of an internal cutoff with ratios ≥1.0 defined as positive by the manufacturer; LMIC – low- and middle-income countries; NAT – nucleic acid amplification testing; POC – point-of-care.

^b^Data from Cheryl Johnson, co-author.

The effectiveness of AHI diagnostics will depend on laboratory complexity, costs, and a range of implementation considerations. Ideal AHI diagnostics will provide reliable and rapid, preferably same visit results and would be easy to use by lay providers requiring minimal additional training for those versed in 3^rd^ generation RDT. Use of more sensitive tests that shorten the window period are essential to confirm HIV-negative status prior to initiating PrEP as false-negative results risk development of drug resistance [17,73–76].

#### Diagnostic algorithms for AHI

Optimal algorithms must balance the consequences of missed diagnoses, false-positive results, and algorithm ease-of-use. Many updated HIV testing algorithms combine 3^rd^ and/or 4^th^ generation assays with more sensitive nucleic acid testing (NAT) technologies [12,67]. NAT has been used successfully for AHI screening in LMICs, even using finger stick specimens [32,69,77–85]. However, in large part due to the resource and logistical limitations, there have been no NAT or 4^th^ generation assay programmes integrated as routine practice in LMICs.

In some populations (e.g., STI patients, MSM), screening for AHI with NAT is cost-effective [80,86–88]. Among MSM in the US, 3^rd^ generation testing followed by NAT was cost saving compared to alternative algorithms [23], and NAT testing alone may be cost-effective [89]. Combining multiple samples for pooled NAT may be a cost-effective alternative compared to testing individual specimens [77,90–92]. In Thailand, the addition of pooled NAT following 4^th^ generation testing improved AHI detection rates, increasing AHI diagnosis by nearly 40% [68,93]. Importantly, pooling strategies also increased screening costs by 22% [68]. These outcomes emphasize that ideal testing algorithms vary according to AHI prevalence, laboratory capacity, and screening volume. Some programmes have elected to recommend repeat testing for higher-risk, seronegative persons, though this approach fundamentally misses AHI diagnoses.

#### Point-of-care testing pipeline for AHI

Both NAT and 4^th^ generation EIAs necessitate relatively sophisticated laboratory infrastructure, venipuncture whole blood, and patient follow-up. Although centralized laboratory testing has been implemented for other diagnostic and monitoring strategies in LMICs, including early-infant diagnosis and VL monitoring, centralized testing interferes with the rapid turnaround time preferred for AHI diagnosis and intervention. The Alere® Determine HIV-1/2 Ag/*Ab* “Combo” device is one of the most studied 4^th^ generation point-of care (POC) technology on the market, and has recently received WHO prequalification status [94]. However, extensive field testing in both the UK and sub-Saharan Africa has demonstrated poor p24 detection rates and frequent false-positive results [95]. A pooled analysis of Determine Combo p24 antigen data from 17,000 participants showed poor sensitivity and high specificity [96–100]. Although newer versions of the Combo assay suggest improved p24 detection on stored specimens, field testing will be critical to confirm performance [101].

The Bio-Rad Geenius™ HIV 1/2 supplemental assay, an immunochromatographic rapid test, measures multiple antibodies and antigens and may simultaneously confirm infection and diagnose early post-antibody conversion acute infections, thus lengthening the diagnostic window for AHI [102]. Other promising POC NAT devices include the Alere Q® [103] (now with WHO prequalification status and validated for RNA detection among infants and children [104]), the SAMBA® semi-quantitative assay [105], the Liat® HIV Quant VL assay [106], and the Xpert® HIV-1 Qual assay [107,108]. Designed to run on the GeneXpert® platform, the dual HIV and TB-disease identification capacity of the Xpert® make it especially appealing given GeneXpert® existing and expanding presence in sub-Saharan Africa, and may be more sensitive and specific for diagnosing AHI compared to other diagnostics under evaluation [108]. Although none designed specifically for AHI screening, the various platforms’ capacity to detect HIV RNA suggests potential for future screening applications [71]. Ultimately, effective and affordable POC tests are needed for effective widespread AHI screening.

#### Clinical and symptom-driven risk-scores for AHI screening

Approximately 29–69% of persons with AHI may seek healthcare for symptoms immediately following HIV acquisition [109,110]. International guidelines have not established a diagnostic strategy for detecting persons with AHI [15]. In LMIC, prioritizing patients presenting with nonspecific symptoms related to acute retroviral illness, such as fever or gastrointestinal distress, may be one approach to optimize screening [110–113]. Unfortunately, AHI can be confused with malaria in many regions of Africa. In surveys of healthcare seeking febrile adults in Uganda [78] and Kenya [114], the prevalence of AHI was greater than 1% and in some clinics was similar to that of malaria [114]. Symptoms – including fever, rash, or diarrhoea – generally emerge approximately 2 weeks following HIV infection [32,115]. Among those presenting with symptoms, the number of symptoms correlates with higher pre-seroconversion peak plasma VL [116], and symptoms tend to cluster immediately preceding or during the period of peak viremia [32]. Although many patients with AHI do not develop symptoms [32], symptomatic patients who seek care during AHI present an opportunity for detection and immediate treatment initiation [117], and may identify persons with higher peak VL [116], and higher VL set point [32,118].

**Figure 1.**
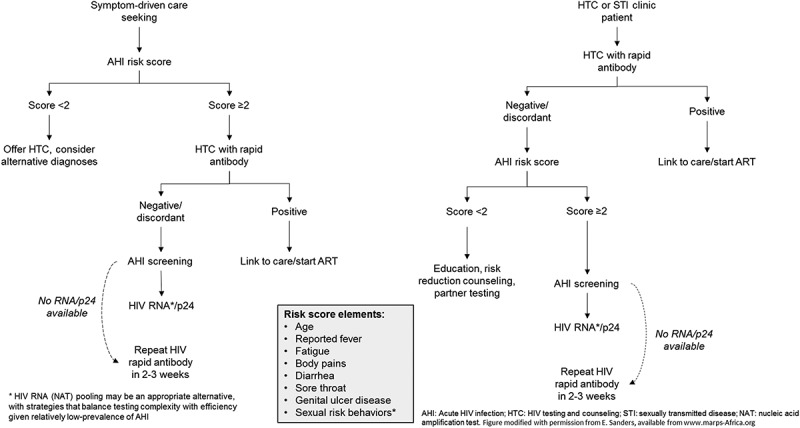
Algorithm for targeting acute HIV screening resources using clinical risk score.

Risk-score algorithms concentrate scarce testing resources with substantial potential cost savings [82,119–121]. Screening patients who meet predefined algorithm thresholds (5–50% of all patients) may identify the majority (>80%) of persons with AHI [119]. However, performance of risk scores vary according to the prevalence of illness presenting with similar symptoms (e.g., malaria), the clinical setting (e.g., STI clinic), and the accuracy of reported symptoms or behaviours (Figure 1) [24].

### Treating persons with AHI

**Figure 2.**
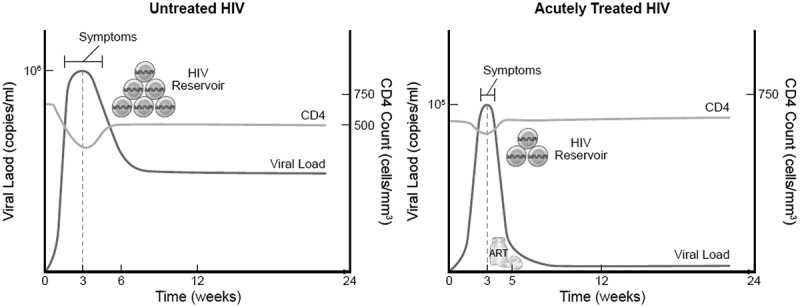
Projected viral load, CD4 cell count, reservoir seeding, and symptom duration comparing untreated vs. treated acute HIV infection.

Updated WHO guidelines recommend all HIV-infected persons receive ART, but make no recommendation about optimal regimens for those with AHI [15]. Treatment during AHI reduces viral reservoir seeding, limits viral diversity, preserves immune responses, and decreases chronic residual inflammation (Figure 2). The early interference with establishment of viral reservoirs may also have important implications for viral eradication efforts. Interruption of therapy initiated early during AHI may result in temporarily lower VL set point, delayed need for restarting ART [122], and, in some cases, sustained virological remission off therapy [123,124]. However, realizing these benefits requires very early ART initiation and may necessitate therapeutic regimens not currently widely available in LMIC.

#### Inflammatory and immune benefits of ART initiated during AHI

ART initiated during AHI may drastically change the course of infection. The seeding of viral reservoirs occurs soon after infection and poses a major barrier to cure efforts [125]. Viral reservoirs are a collection of cells harbouring virus despite suppressive therapy. ART initiated during the first two weeks of infection is the most effective strategy for limiting size of latent reservoirs [20,126–130]. Early ART reduces overall level of proviruses, and thus the number of intact, potentially transmissible, proviruses [131], preserves mucosal CD4 T-cell counts, and reverses inflammation in major anatomical reservoirs including gut and lymph nodes [132,133]. Early ART also reduces the number of infected cells, lowering cell-associated HIV DNA levels [130] and, in doing so, reducing the viral set point [134]. Conversely, ART initiated during chronic infection results in larger reservoirs, and contributes to rapid viral rebound with ART interruption [135–137].

ART initiation during AHI also results in improved HIV-specific immune responses, potentially slowing disease progression [124]. CD4 T-cell depletion begins shortly after infection and delaying ART compromises immune system recovery [138–141]. CD4 cell counts and CD4/CD8 ratios predict long-term morbidity and mortality [142,143]; CD4, CD8, and CD4/CD8 ratios are more likely to normalize with treatment started during AHI compared to delayed treatment [141,144–147], with CD4 cell counts normalizing within one year [148]. Very early ART (within 40 days of the estimated date of infection) is associated with a significant increase in the CD4/CD8 ratio compared to delayed ART [149]. ART initiated during AHI is associated with lower T-cell activation levels at 48 months compared to ART initiated during chronic infection, improving immune-system recovery and potentially decreasing mortality [138]. Lifetime durability of these responses is unknown, although retrospective analyses suggest sustained reactions for up to five years [141].

#### Ideal ART regimens for AHI

In the era of expanded ART options, another consideration is what regimen is appropriate to start during AHI. Observational and randomized studies comparing standard ART to “intensified” regimens that include integrase or entry inhibitors consistently demonstrate similar effectiveness in raising CD4 T-cell counts, and lowering markers of immune activation and cell-associated DNA [150–153]. Although intensified regimens may not be necessary to reap virological or immunological benefits, intensified regimens result in a more rapid VL decline in blood and genital secretions [154]. Rapid VL reduction during AHI is critical to interrupt transmission, and is best facilitated by use of integrase inhibitors [155,156]. Other factors to consider in regimen selection include tolerability (i.e., toxicity), resistance (both transmitted and genetic barriers to acquiring mutations), and financial implications. Importantly, current WHO treatment guidelines do not include the newer entry inhibitors. Ultimately, in the setting of safe and potent ART options, the health consequences of not treating persons with AHI likely outweigh the risks inherent to withholding treatment.

### Public health implications of AHI diagnosis and treatment

AHI accounts for a small, but nontrivial portion, of all new HIV diagnoses – identifying an additional two to 10% of HIV above antibody-only testing depending on risk profile of screened patient population (i.e. STI clinic vs. general outpatient setting) [79,83,84,90,157–161]. Insufficient attention to AHI-centric public health interventions could partially explain the stagnant global HIV incidence trends: primarily driven by LMIC in sub-Saharan Africa, and with significant heterogeneity by region, stable incidence despite increases in treatment coverage towards UN 90–90–90 treatment goals over the past five years reinforce inadequacy of current screening strategies [162–164].

#### PrEP in context of undiagnosed AHI

Missing persons with AHI could also have substantial consequences for PrEP programmes. WHO guidelines recommend PrEP for HIV-negative persons at substantial risk of HIV infection [15]. There is likely substantial overlap in the population that would be eligible for PrEP and those who are at greatest risk of AHI [165]. Given that PrEP formulations and WHO-recommended first-line therapy share a common tenofovir-containing backbone, induced resistance selected for by PrEP administration to persons with unrecognized AHI could have significant public health implications. Although induced resistance may decay following therapy interruption [166], these mutations must be accounted for in future ART selections. However, mandating complex AHI testing algorithms prior to PrEP initiation could impede PrEP implementation.

#### Public health interventions targeting AHI partners and behaviours

AHI screening enables partner notification services and behavioural interventions. AHI screening has proved feasible in several low-income [77,79,84], middle-income [32], and high-income settings [24,65,90]. Despite limited data regarding feasibility of AHI screening outside of clinical research settings, studies suggest that testing is both acceptable and effective. Implementation research is critical to tease out setting specific implementation barriers to more widespread AHI detection and treatment.

Assisted partner notification, including provider-initiated partner notification, is a proven, safe, and cost-effective intervention for identifying persons with undiagnosed infection [167–172], although programmes are rarely implemented in LMIC. AHI diagnosis may facilitate more effective partner services. Persons with AHI name twice as many sex partners in the three months preceding diagnosis compared to persons newly diagnosed with chronic infection [173,174]. In the context of more recent infection, named partners are also more likely to be infected with HIV [173,175], although many of these source partners may have been previously diagnosed [176].

AHI is often diagnosed during a period of increased sexual risk, suggesting the importance of rapid behavioural interventions [177–179]. Given that persons with AHI can name more recent sex partners who are respectively at greater risk of HIV, this provides an opportunity for behavioural interventions among serodiscordant couples [180] or serosorting [181]. The feasibility of behavioural interventions is complicated by the need for programmes to rapidly change behaviour in the setting of increased sexual risk activities, anxiety, depression, and substance abuse [181–184]. Despite perceived changes in behaviour, many with AHI are unable to abstain from sex or use condoms, particularly with long-term partners [185]. Incident sexually transmitted infections after diagnosis suggests ongoing risk behaviours despite risk reduction counselling and retention in care [186]. The appropriate duration and intensity of behavioural interventions is unknown, although it seems that modified post-test counselling can adequately convey messages of increased infectiousness and possibly facilitate behaviour change in the short term [177,178].

#### Unique challenges and opportunities in treating AHI

Along with appropriate counselling, early ART initiation during AHI is critical to limit the spread of HIV attributable to persons with AHI [9,187]. High viral loads are a modifiable risk factor for HIV transmission [3]. Same-day ART initiation for persons with AHI, in line with current *Treat All* initiatives, results in more rapid viral suppression compared to non-same day initiation [188]. ART in AHI is highly effective and viral failure is uncommon [189]. While virological response for persons with chronic infection is characterized by suppression 6 months after ART initiation, the most effective means of reducing transmission during AHI requires regimens that rapidly reduce viral burden in genital fluids. Among Thai MSM with AHI, comparing a 3-drug regimen (2 NRTI + 1 NNRTI) to that same regimen intensified with entry and integrase inhibitors (5 drug regimen, including raltegravir), persons on the 5-drug regimen achieved viral suppression in seminal fluid faster compared to standard regimen (13 vs. 24 days); this period of sustained viremia may represent a critical window of ongoing transmission [154]. Although not currently included in most first-line schedules in LMIC, the more rapid viral load decline with inclusion of an integrase inhibitor agent make this an important regimen modification to reap potential transmission benefits of early ART during AHI. Ultimately, data demonstrating the feasibility and effectiveness of early ART in suppressing viremia and interrupting transmission suggest that appropriately-implemented treatment-based interventions may change the course of the global epidemic.

Viral response depends on regimen and patients’ adherence behaviours. For example, the 24-week failure rate among a Thai cohort (1.1%) was significantly lower than the 10% failure rate among a Boston-based cohort also receiving early-ART [190]. The difference may be related to the differences in regimen selection, with the Boston study demanding a higher pill burden. Despite the potential suppression benefits of more potent regimens, compared to three-drug combinations, adherence to the intensified five-drug regimen is generally worse [150,151], likely related to the necessary twice-daily dosing and more frequent adverse effects. Although more complex regimens may be preferred for rapid reduction of VL, the adverse effects, risk of sub-standard adherence, and drug costs could prohibit widespread use.

#### System-wide barriers to AHI diagnosis and treatment scale up

Existing public health systems are poorly equipped to deal with AHI detection, treatment, and linkage to prevention and care. The potential to stop transmission during AHI using partner tracing, behavioural, or biomedical interventions relies on rapid diagnosis and ART initiation. Barriers to AHI diagnosis include complexity and expense of diagnostics, limited training among health professionals to implement diagnostics on a wide scale, and the relative brevity of the infection stage [191]. Increased HIV testing by lay providers has substantially improved access to HIV testing services [16]. Unfortunately, even 4^th^ generation assays require non-capillary whole-blood or plasma specimens, and thus may not be ready for scale up. Point-of-care tests are critical for expansion of AHI screening, and there are promising alternative platforms under development and in early field implementation. However, cost, ease-of-use, adequate sensitivity for screening objectives, and efficient throughput for high-volume screening are still under investigation. In the absence of widely available diagnostics capable of detecting AHI, one strategy includes encouraging retesting 2–4 weeks after negative or inconclusive antibody results for persons with acute retroviral symptoms at time of presentation. Unfortunately, over 40% of participants failed to return for follow-up testing even after enhanced appointment reminders using SMS or in-person cues [192].

Barriers to diagnosis and treatment are intimately intertwined. Therapy initiated within two weeks of infection may be most beneficial for reduced transmission and improved immune response. However, this narrow window requires extremely sensitive diagnostic assays and system capacity to start ART almost immediately after a positive result. Although rapid and reliable diagnosis is the critical first step for AHI intervention, comprehensive management will also require appropriate provider messaging, patient education, and possibly AHI-specific treatment strategies.

## Conclusions

AHI detection and treatment has important diagnostic, clinical, and public health implications [193]. Treatment is beneficial for the infected individual, with likely long-term immune-virological benefits with early treatment initiation, and extensive research has demonstrated advantages of early ART, regardless of regimen. Immediate ART is also advantageous to uninfected sex partners, reducing the risk of acquisition in the setting of suppressed viremia. Furthermore, immediate ART during AHI has profound implications for HIV cure efforts; by eliminating or limiting establishment of latent reservoirs and preserving immune responses, early ART during AHI may be the key to ongoing eradication strategies [194]. Although, currently, treatment interruption is an unfavourable approach for AHI management, the development of predictive algorithms with HIV eradication objectives, particularly in LMIC, will need to take into consideration parameters that may influence control of infection [195,196], such as route of infection, ethnicity, and hormonally driven gender-based differences [197,198], including evidence of lower rates of inducible virus among women on suppressive ART [199]

We identified several research priorities. First, we need prospective assessment of appropriate testing strategies and algorithms for AHI diagnosis, including evaluation of risk score performance for screening in LMIC using same-day POC tests. This is important for implementation and policies related to scaling up AHI detection and treatment as well as PrEP implementation in LMIC. Second, further diagnostic research on POC assays to detect early and acute infection is key. Third, we need investigations into the feasibility, cost-effectiveness and implications for treatment switch algorithms associated with incorporating integrase inhibitors into first-line regimen recommendations. Use of integrase inhibitors will be especially important among certain high-risk groups for whom rapid reduction in viral load is critical, such as pregnant women. Finally, implementation research in the context of expanded testing and treatment goals will become increasingly important to understand how to incorporate routine AHI detection and treatment into existing health systems.

The outlined research agenda is essential for guiding policy change. Nonetheless, we propose that LMIC with AHI testing capacity should immediately implement AHI screening among high-risk populations. Targeted screening utilizing risk score algorithms may help identify persons with AHI and focus limited resources without confining screening to specific clinics. Additional approaches include social and sexual partner recruitment and notification to help increase screening yields. Scaling up testing requires extensive personnel training and patient education and should be pursued in settings in which immediate ART as recommended through the *Treat All* policy can be offered.

This scoping review addresses the broad topic of clinical and public health implications of AHI. As a scoping review, we did not define inclusion nor exclusion criteria and, as such, we cannot assess the quality of published articles that are included in the review in a standardized fashion. Unlike systematic reviews, scoping reviews such as this do not synthesize evidence in a manner that allows relative weight of alternative research findings or interventions, offering instead a narrative from which future systematic reviews may be motivated. Despite these limitations, the scoping review provides flexibility and breadth in study design and publication inclusion on a fast-changing and, specifically in LMIC, under-researched topic.

Unchanged HIV incidence rates despite ART scale-up suggest the importance of targeting diagnostic and treatment interventions to groups responsible for a disproportionate share of new infections, including persons with AHI. Although current WHO guidelines recommend a *Treat All* approach, explicit guidance regarding AHI testing and treatment approaches are urgently needed. Further AHI research and policy development can help to fill this gap.
